# Human development and reproduction in space—a European perspective

**DOI:** 10.1038/s41526-023-00272-5

**Published:** 2023-03-27

**Authors:** Varsha Jain, Susana M. Chuva de Sousa Lopes, Mohammed A. Benotmane, Vittore Verratti, Rod T. Mitchell, Jan-Bernd Stukenborg

**Affiliations:** 1 MRC Centre for Reproductive Health, The Queen’s Medical Research Institute, The University of Edinburgh, Edinburgh, UK; 2 Department of Anatomy and Embryology, Leiden University Medical Center (Building 2), Leiden, The Netherlands; 3 Radiobiology Unit, Belgian Nuclear Research Centre ‘SCK CEN’, Mol, Belgium; 4 Department of Psychological, Health and Territorial Sciences, “G. d’Annunzio” University, Chieti-Pescara, Chieti, Italy; 5 Royal Hospital for Children and Young People, Edinburgh, UK; 6 NORDFERTIL Research Lab Stockholm, Childhood Cancer Research Unit, Department of Women’s and Children’s Health, Karolinska Institutet, and Karolinska University Hospital, Solna, Sweden

**Keywords:** Risk factors, Developmental biology

## Abstract

This review summarises key aspects of the first reproductive and developmental systems Science Community White Paper, supported by the European Space Agency (ESA). Current knowledge regarding human development and reproduction in space is mapped to the roadmap. It acknowledges that sex and gender have implications on all physiological systems, however, gender identity falls outside the scope of the document included in the white paper collection supported by ESA. The ESA SciSpacE white papers on human developmental and reproductive functions in space aim to reflect on the implications of space travel on the male and female reproductive systems, including the hypothalamic-pituitary-gonadal (HPG) reproductive hormone axis, and considerations for conception, gestation and birth. Finally, parallels are drawn as to how this may impact society as a whole on Earth.

## Introduction

Humans have been travelling into space for over 60 years and to date, human conception in space has not occurred despite much public and media interest in the subject. Space impacts human physiology at every fundamental level, whether this is due to the microgravity environment, the exposure to higher radiation doses compared with being on Earth, the change in circadian rhythm experienced during spaceflight or the stress of space travel itself. The overall physiological and psychological adaptation to the spaceflight environment is similar for male and female astronauts; however, there are subtle differences detected in almost all body systems due to sex and gender. Understandably, the male and female reproductive systems, as they are entirely different, deserve independent focus and research to understand the short and long-term impacts of the spaceflight environment. So far, there has been limited research into reproductive physiology in relation to space travel. The ESA SciSpacE white papers set out the research that will be needed to advance our knowledge in this important field of space physiology in order to support advances in space travel and habitation by humans^1^. In this review, we briefly summarise the principal aspects of human development and reproduction, and relate them to studies which can be performed under space-relevant conditions.

## Key knowledge gaps of reproductive function and human development in space

Currently, there is limited knowledge available on the systemic effects of spaceflight stressors, e.g. altered gravity (micro-, hypo- and hypergravity), increased radiation, social isolation, confinement, sleep disturbances, dietary changes and any associated stress/anxiety on the hypothalamic-pituitary-gonadal (HPG) axis in females and males and how these stressors impact the functionality of the reproductive organs^2^. Due to the small number of astronauts who have undertaken long duration spaceflight, it has not been possible to determine whether spaceflight stressors or space radiation impact fertility, versus the ageing impact which naturally affects reproductive outcomes^2–4^. It is therefore important to gain knowledge on how increased and prolonged space radiation (for short and long periods) affect the functionality of female and male reproductive organs, as well as the overall impact on cancer risk, not only for the astronaut, but also their progeny and for future generations via effects on their gametes. We highlight the key knowledge gaps, which apply for female and male reproductive systems, conception, embryo development and birth (summarised in Table 1).

**Table 1:** Potential priorities for future space programmes as described in the white paper collection of ESA according to previously identified key knowledge gaps, with a focus on microgravity and/or exploration relevance^a^.

**Physiological changes related to human reproduction and development**	
• ‘Sex’/‘Gender’ as well as sex steroid hormones in biological systems.	
• Teratogenic and oncogenic space related-effects on reproductive systems.	
• Foetal development of body systems in space-simulating conditions.	
• Age-dependent evaluation of late memory, learning and cognitive performances.	
• Long-term effects on gestation under space conditions.	
• Monitoring development and function of offspring conceived and developed in space.	
**Male reproductive health aspects**	**Female reproductive health aspects**
• Spermatogenesis relies on testicular spermatogonial stem cells, which require a functional stem cell niche.	• Hypothalamic-pituitary-ovarian-endometrial interplay is tightly regulated for normal functioning
• Generation of functional sperm, requires spermatogenesis, spermiogenesis, and final maturation processed in the epididymis.	• Quantification of venous thromboembolism risk to female astronauts using the combined oral contraceptive pill.
• Androgens are essential for development and function of the internal and external reproductive organs	• Multidisciplinary professional teams are crucial in providing pregnancy and neonatal care.
**Timeline of potential research questions regarding reproductive health aspects**	
Short term	• How is the menstrual cycle, including age of menopause, influenced by spaceflight?
	• What is the impact of spaceflight on quality and competence of gametes (eggs/sperm)?
	• How does space travel impact gynaecological conditions in female astronauts?
	• What is the effect of space travel on libido and sexual attraction?
Medium term	• How does prolonged exposure to space radiation affect HPG axis regulation in adults?
	• How does menstrual suppression affect female astronauts?
	• What are the effects of prolonged exposure to the space environment on reproductive organs?
	• Can a multidisciplinary team be developed to support pregnancy and birth in space?
	• What is the role of hormone replacement therapy in female astronauts?
Long term	• How does prolonged exposure to space radiation affect HPG axis during puberty/aging?
	• Are there transgenerational effects on gamete quality?
	• Is medical assisted reproduction feasible in space?
	• Do female astronauts need additional monitoring during a pregnancy after space travel?
	• What are the effects of spaceflight during the early postpartum phase?

Timeline: short [3 years], medium [5 years], long [10 years]).

^a^Ref. ^1^.

### Principles of the male reproductive system

The majority of astronauts/cosmonauts are men. However, very little is known about the effects of spaceflight on their fertility. Fertility in males is dependent on the presence of a germ cell population, which migrate into the developing testis during the first trimester of pregnancy. During pregnancy, the foetal germ cells undergo epigenetic reprogramming and many are actively proliferating, making them susceptible to radiation. These foetal germ cells give rise to a pool of spermatogonial stem cells (SSCs) that are capable of differentiation, meiosis and development into sperm in adulthood. In adulthood, a continuous supply of sperm is achieved by maintaining a balance between SSC differentiation and self-renewal. However, the ability of SSCs to produce sperm is also dependent on the presence of functional somatic cell populations, such as supporting Sertoli cells and testosterone-producing Leydig cells. The function of these cell populations is primarily regulated by the HPG axis^5^. Puberty in males is initiated from about 9 years of age and at ~13 years of age, the process of spermatogenesis results in the production of sperm (Tanner stage 4). The unique testicular microenvironment, that includes SSCs and supporting somatic cell populations, plays a crucial role regulating the levels of specific growth factors and hormones. This makes the pre- and postnatal testis vulnerable to disruption by environmental factors such as environmental toxins, radiation, and alterations in gravity (e.g. micro-, hypo- and hypergravity) in space and on Earth^4,6–9^.

### Space and the male reproductive system

The majority of studies conducted to date have used rodents and focused on the impact of altered gravity on spermatogenesis and testosterone synthesis in adulthood, in addition to radiation-related impairment of germ cell maturation and differentiation^10–12^.

Two recent studies, reported the effects on male reproductive functions in male mice housed on the International Space Station (ISS)^13,14^. In 2019, Matsumura and colleagues investigated the effects of microgravity on the reproductive function in male mice under microgravity and artificial gravity (around 1 G) on the ISS, compared to mice housed on Earth as ground controls^13^. The study showed that adult male mice housed on the ISS for 35 days showed, when back on Earth, no adverse effects on sperm quality or on the viability of their offspring^13^. However, in a more recent study, Yoshida and colleagues reported epigenetic alterations in sperm of adult mice housed in space (on the ISS) for 35 days^14^. Here, dysregulation of 24 genes (19 upregulated and 5 downregulated) was observed in the liver of the progeny, although functional abnormalities in the liver of the offspring were not reported. Additional to alterations in small RNA expression in spermatozoa, alterations in binding of the transcription factor ATF7, which binds to promotor regions in male germ cells and induces methylation of histone H3 Lysine 9 (H3K9me2) suggested an intergenerational effect^14^. It can be speculated that the increased production of reactive oxygen species (ROS) as well as the epigenetic alterations induced by environmental stressors (e.g. cosmic radiation) may contribute to dysfunction in physiological pathways underlying HPG axis, seminal profile, and erectile function, leading to impaired male fertility. To minimise damage to sperm by such stressors, the use of frozen-thawed sperm for reproductive purposes could be one option. In a recent study, birth of healthy mouse pups was obtained following intra-cytoplasmic sperm injection (ICSI) using frozen-thawed sperm stored for up to 5 years and 10 months on the ISS^15^. This follow-up study from a previously reported study by the same research group^16^, revealed that long-term storage of frozen-thawed sperm in space is a potential option to store mammalian sperm for future use^15^. However, future research is warranted to elucidate the details behind (epi)genetic alterations reported as well as the impact on humans.

It is well recognised that male reproductive development is programmed in foetal life, and is highly androgen dependent^17^. Key events in postnatal life such as the mini-puberty of infancy, testicular maturation during childhood and puberty are also essential for ensuring a healthy male reproductive system in adulthood. So far, the impacts of gravitational changes and radiation on reproductive function from foetal life to (peri)puberty have not been investigated. The same applies to detailed studies of effects in space beyond the low Earth orbit (e.g. on Mars), on SSCs and stem cell niche functions as well as germ cell maturation, epigenetic profile, sperm quality and quantity in humans, and therefore warrant future studies exploring potential harmful effects of space travel on male fertility.

### Principles of the female reproductive system

According to the statistics published online (www.worldspaceflight.com/bios/stats1.php), as of November 2022, 639 individuals have travelled into space, out of which, 72 were female astronauts or cosmonauts, a mere 11%. Females begin puberty from approximately 8 years of age and on average puberty completes when girls experience menarche (first period) between the ages of 12–14 years (Tanner stage 4)^18^.

### Menstrual cycle physiology

Menstruation occurs in a cyclical manner, via a delicate balance of hormonal stimuli, governed by the HPG axis. The predominant hormones which influence menstruation within this axis are gonadotropin releasing hormone (GnRH), follicle stimulating hormone (FSH) and luteinising hormone (LH), released by the brain, as well as oestradiol (E_2_) and progesterone (P_4_) secreted by the ovary^19^. A repetitive cycle of scarless tissue injury and repair occurs within the dynamic endometrium; this involves proliferation, decidualisation, inflammation, apoptosis, haemostasis, vasoconstriction, hypoxia, repair and regeneration^20^. The ovarian cycle runs in parallel to the menstrual cycle of the uterus (the ovarian follicular phase is synchronous with the endometrial menstrual and proliferative phase; the ovarian luteal phase is synchronous with the endometrial secretory phase).

### The female reproductive life course

In females, the reserve of primordial follicles is determined during embryonic development and these diminish continuously in number and quality with age^21^. The average age when female fertility begins to decline is 32 years on Earth^22^. The average age of menopause is 51 years and this signals the cessation of the reproductive organs, in particular the ovary, functioning for the purposes of maintaining a pregnancy^23^. Prolonged exposure to radiation (e.g. due to prolonged space travels) may accelerate the decline of the follicular pool and result in premature ovarian failure and an earlier onset of menopause^24^.

### Space and the female reproductive system

Extreme states of stress can impact the menstrual cycle on Earth^25^; however, it is unknown whether this would have the same impact on natural menstrual cycles in space. Data from female astronauts who flew during the Shuttle era suggest that pregnancy rates and associated complication rates, are equivalent to ground-based, age-matched controls^26^. Female astronauts on long duration missions predominantly use the combined contraceptive pill (COCP) to suppress menstruation^27^ and there has been so far no reported impacts on fertility rates compared to age-matched controls on Earth. Due to the high proportion of female astronauts using the COCP for menstrual suppression, is remains unknown whether the spaceflight environment impacts endometrial or ovarian functioning^3,4^. For example, it is unknown whether the immune changes experienced during spaceflight have an impact on the immune cell populations within the endometrium, or whether the vasculature within the endometrium behaves in an altered manner with long-term exposure to spaceflight and therefore predispose the female astronaut to abnormal bleeding patterns. Finally, with the first episode of venous thromboembolism (VTE) in space reported in 2019^28^, it would be important to further investigate risk factors^29^ which may impact female astronauts taking the COCP, a known risk factor for VTE^30^.

There is limited data on the impact of spaceflight stressors on the menstrual cycles in humans and animals; however, the simulated model of menstruation in mice provides a potential avenue for further research^31^. Moreover, the effects of increased space radiation for a prolonged period on female reproductive organs such as uterus, cervix, fallopian tubes, ovaries (which contain of the entire follicular reserve) and breasts are not well studied, due to existing recommendations from national committees on radiation safety that limit exposure^32^. However, it is well known from patients undergoing cancer treatment (radiotherapy), that oocytes are very sensitive to radiation^33^. The overall quantity and quality of oocytes (folliculogenesis takes ~50 days in mice^34^ and 6 months in humans^35^) would also need further investigation from a fertility perspective. In this regard, it would be important to conduct long-term follow-up studies as a potential new platform, to investigate female reproductive health both during spaceflight and upon return to Earth, with comparisons being made with ground-based, age-matched controls. This would allow both medical and research data to be collected for both humans and non-human mammals, allowing a retrospective comparison.

### Sex differences in space

It is important to define the terms sex and gender as they are often used interchangeably, however, they have different meanings, and therefore different implications when planning scientific research in space. The World Health Organisation defines “gender” as the socially constructed characteristics of women and men, whereas “sex” refers to biologically and physiologically determined characteristics (https://www.who.int/health-topics/gender). Almost all body systems are impacted in their adaptation to space^36^, hence it will be important to ensure all human research roadmaps include at least both male and female astronauts in order to tease out sex differences. Sex steroid hormones (oestrogen, progesterone and androgens) have an impact on multiple body systems and therefore when planning studies including females, it would be prudent to note the menstrual cycle stage, as hormone levels can vary, and this could impact the results (e.g. musculoskeletal conditioning at varying time points during the menstrual cycle may be altered)^37^. One example of sex-based differences relevant to spaceflight is the reduced incidence and severity of spaceflight associated neuro-ocular syndrome (SANS) in female astronauts compared to male astronauts^38^; investigating this difference and why it occurs is of importance to prevent long-term implications for all astronauts in the future. As the general population is ageing and on average ISS-astronauts are older than Shuttle era astronauts^29^, changes associated with ageing in space, including the post-menopausal years would also be important to monitor. Female astronauts are more susceptible to radiation-based cancers^3^; however, prevention and screening protocols for cancer in relation to astronauts who have been to space, have not been systematically implemented, e.g. would the increased radiation exposure of space necessitate an adjustment to the cervical or breast cancer screening which is usually undertaken by women on Earth?

### From conception to birth

Pregnancy is contraindicated during spaceflight and there has been no recorded episodes of human conception occurring in the spaceflight environment. However, in view of a permanent Moon station or the possibility of colonising other planets (e.g. Mars), this important aspect of human life in space needs to be considered alongside its associated challenges.

Conception starts when a sperm cell penetrates a competent mature oocyte forming the zygote. The zygote develops as it travels from the fallopian tube to the uterus^39^. When inside the uterus, the embryo, known as a blastocyst, hatches and implants in the primed and decidualised endometrium, the inner layer of the uterus. During the following months, the placenta becomes the interface where maternal blood comes into close proximity with foetal blood, providing nutrients and gases to the developing foetus, whilst removing waste products^39^. During the first trimester, the foetus undergoes organogenesis, with sex determination and the formation of the reproductive organs. This is especially critical in the period between 6- and 14-weeks post-conception^40,41^. During organogenesis, the embryo is sensitive to environmental stress (chemical, physical and viral infections), which may induce malformations or congenital abnormalities^42–44^. Effects of radiation on brain development have been illustrated in atomic bomb survivors of Hiroshima and Nagasaki in Japan showing high rate of malformations (microcephaly) and decreased intelligence quotient (IQ) in children that have been exposed in utero in the first trimester between week 8 and 15 of gestation (equivalent to 6–13 weeks post conception)^45,46^. Mice exposed to radiation during the critical period of neurogenesis and brain formation (between embryonic day E10 to E13) showed increase in apoptosis, neuroinflammation and premature neuronal differentiation^47–49^, which are most-likely the major causes of microcephaly and late behaviour defects. The first trimester is also the period when miscarriage rates are at their highest, which may be due to environmental or genetic causes, with the specific causes frequently unknown. The maternal circulation opens to the placental (intervillous) space at the end of the first trimester^50^, when most organs enter a period of pronounced growth until birth.

The average gestation period is about 37–42 weeks^51^. Labour and delivery can be associated with life-threatening situations for both mother and child, for example, acute haemorrhage in the mother during or after delivery or prolonged second stage of labour, delaying delivery and increasing the risk of hypoxic ischaemic encephalopathy^52^. It may be vital to receive acute medical treatment, such as a caesarean section, a blood transfusion or maintaining the newborn in a neonatal intensive care unit. The first 6 weeks postpartum are critical for both mother and child. The maternal hormonal levels readjust to pre-pregnancy levels and the uterus remodels to return to its pre-pregnancy size. Within the first minute of life, the baby must start breathing, maintain its own circulation, oxygenate its vital organs and show signs of life. Usually, mother and baby have early skin-to-skin contact, which is important for bonding, to regulate the body temperature of the baby and to encourage the let-down of breast milk^53,54^. The baby will require feeding almost immediately post-birth and this can usually be commenced via breastfeeding. A multidisciplinary team of professionals may be necessary to keep mother and baby healthy during this time; one which is not readily available in space.

### From conception to birth in space

The main safety risks for a pregnancy in space, are not only the effects of excess radiation on the health of the foetus and future baby but also the effects of space radiation on the developing germ cells (future gametes of the baby) that will be used to produce the future generations. There is potential risk to their functionality and quality, directly influencing the health of the second generation^14,55,56^. During mid-gestation, the developing germ cells undergo epigenetic reprogramming as well as meiotic recombination (exclusively in female foetus), hence it will be important to gain knowledge on the effects of spaceflight stressors on the future fertility of foetuses (starting with transgenerational animal studies in space). The scarce data available on rodent pregnancy and delivery rates report high resorption and mortality both as stillbirth and during the first postnatal week. However, the pups that survived to adulthood were able to conceive and carry viable litters^57,58^.

Regarding the health of the foetus itself, it has been shown that exposure to radiation during gestation is considered highly harmful to the development of the foetus^59^. Radiation doses equivalent to a total dose incurred during a 3-year Mars mission would lead to structural malformations in human embryos, including, microcephaly and microphthalmia as well as neural tube defects^60^. Effects on central nervous system (CNS) formation have been shown to lead to functional brain defects resulting in low IQ and mental health impairment^61^. Ground-based animal experiments confirmed the teratogenic and behavioural effects of radiation at different stages of gestation. Exposure during early organogenesis (embryonic day E7.5) in mice was shown to lead to more structural malformations (exencephaly, micropthalmos/anopthalmos)^62^ with low survival rate at 1.0 Gy X-rays, while exposure during late organogenesis (embryonic day E10–E13) induces both structural (microcephaly) as well as functional (cognitive impairments) defects^49^.

Embryonic neuron cells cultured under space conditions using RPM (random positioning machine) and simulated radiation showed clear modulation in neuronal plasticity evidenced through morphological and physiological changes. These changes were dependent on the duration of exposure to microgravity. Significant alterations in neurite network, neuron morphology and viability following exposure to simulated space conditions were observed. Prolonged exposure to simulated microgravity (10 days) revealed a high adaptation of neurons to the new gravity conditions as well as a partial adaptation of neuronal networks. However, neurons and neuronal networks exposed for long term to simulated microgravity required longer recovery time to re-adapt to the ground gravity^63,64^. These cellular investigations indicate the harmful effect of combined space conditions (microgravity and radiation) on maturing neuronal cells, altering neuronal survival, connectivity and neural network formation. Thus, normal brain development seems to be disturbed under space conditions, which would compromise brain structure and function at young and adult age.

Interestingly, folic acid food supplementation seems to be a potential radiation mitigator to counteract radiation effects in mice^65^ by reducing significantly the rate of malformations induced by radiation during early organogenesis. Thus, targeted research towards countermeasures and new compounds mitigating radiation effects during gestation is highly required. Systematic investigations should be conducted to understand the underlying mechanisms behind cosmic radiation and microgravity on the developing brain, and identify and develop mitigating compounds for structural changes (malformations) as well as functional (cognitive and behaviour) abnormalities. Moreover, foetal development in space with its elevated growth rate may result in high risk of increased DNA damage and genetic mutations that could lead to abnormalities or even cancer, such as leukaemia^66,67^. In this regard, the use of animal models or in vitro systems (using stem cells or organoids) may shed light into aspects of embryonic development and help characterise the mutational load in space.

Another important aspect that needs further investigation is the development of the maternal-foetal interface in space, especially the period of implantation and placentation and its response to spaceflight stressors including increased radiation. Spaceflight may cause increased oxidative stress, which has been associated with placental dysfunction that could lead to pre-eclampsia, intrauterine growth restriction, preterm birth and gestational diabetes (especially in view of the increased insulin sensitivity associated with spaceflight)^68^. Both implantation failure and miscarriage can be influenced by nutrition, stress-related factors and teratogenic medication. Although one study has reported on spontaneous abortion among female astronauts upon returning to Earth^26^, longer follow-up studies and data collection are necessary. Of high importance during pregnancy is also the additional effects of stress, nutrition and immune system activation. As astronauts are taking longer duration flights, they may be delaying pregnancy until after their missions. It is unknown whether spaceflight increases the risks of cardiovascular complications during pregnancy, e.g. venous thromboembolism or pre-eclampsia, and whether extra monitoring is required during subsequent pregnancies.

Finally, throughout a pregnancy journey, a woman needs the care from a multidisciplinary team as well as tailored support at key time points. Offering these in the space environment poses many challenges and these would need to be examined in further detail to assess whether it would be possible to offer this multidisciplinary care in a remote/telemedicine format.

### Birth and first postnatal weeks in space

Although the thought of childbearing in space has been fascinating mankind for years^69^, only very limited number of studies have reported on the partum and postpartum effects on rodents and the time of housing in space in these available studies has been relatively short (15 days)^13,14^.

It will be important to increase knowledge of the effects of spaceflight in the events associated with the partum and postpartum, as contradictory results have been reported, regarding delivery, wellbeing and subsequent breastfeeding behaviour, with direct consequences to pup mortality^4^. Interestingly, duration of labour, higher number of contractions, low birth weight and higher mortality have been often reported^4^. Moreover, studies on launching rodent pups of several ages with their mothers into space, reported that 5-day and 8-day-old pups showed high mortality^26^.

Studies using pregnant rats exposed to hypergravity (1.5 G) during the second half of pregnancy and first postnatal week, resulted in increased neonatal mortality in the pups of rats experiencing the first pregnancy, but not in the pups of rats experiencing the second pregnancy^70,71^.

Using animal models to increase our understanding of the postpartum period under microgravity and increased radiation on organs in particular the brain, lung, speech and locomotion as well as the limited social interaction and cognitive stimuli will be invaluable if we are to consider the presence of human life for long periods of time in space.

## Benefits for Earth and relevance of studies conducted in space or in space analogue environments

### Female/male reproductive system

Research into the reproductive organs and systems in the spaceflight environment may also provide vital information for Earth-based functioning and vice versa. In vitro conditions to study gametogenesis have the potential to open lines of investigation in space and on Earth for possible differences experienced with ageing, disease, environmental pollutants or gonadotoxic medical treatments, including radiation. Knowledge of the gonadotoxic effects, such as dose-dependent effects of radiation in space, can be beneficial to identify and specify risk factors of current or future medical treatments used on Earth. Developing strategies to generate functional gametes from a patient’s own germ cells (oocytes and spermatogonia) or potentially from non-germ stem cells (e.g. induced pluripotent stem cells) in the laboratory via in vitro gametogenesis, can circumvent failure to generate competent oocytes or sperm naturally. Recently, the combination of culture conditions designed for differentiating germ cells into more matured states, could demonstrate the differentiation of pluripotent stem cells (PSCs) into functional sperm in mice^72^. In 2016, the successful reconstitution of the entire oogenic process in mice was reported^73^. These studies, together with first studies reporting the generation of immature human oocytes and pro-spermatogonia (for review see ref. ^74^), suggest the potential to generate functional gametes from human PSCs in the future, which may offer an alternative source for gamete production, when mature sperm or oocytes generated from natural immature germ cells are not available. Although promising, this technology is not yet available in humans, but would be of benefit to patients on Earth and would represent a major clinical advance with additional benefits to the industry. Should it be possible to generate competent oocytes and sperm during spaceflight conditions, these could be generated in space or in similar conditions on Earth for potential future use.

### From conception to birth

Research that advances how we keep humans healthy in space, has the potential to generate strategies to protect pregnant women and their unborn children on Earth. For example, the identification of compounds to counteract the harmful effects of radiation in space, could allow access to imaging or therapeutic modalities for pregnant women that are currently not possible due to the harmful effects of radiation. These countermeasures could be administered to cancer patients prior to exposure to radiation therapy to mitigate the effect of radiation on healthy tissues. In addition, such countermeasures may improve health and cognition in the general population.

### Partum and postpartum

Understanding the effects of stress factors, ionising radiation and changes in the inflammatory system^3^ during the early postnatal period, could be valuable to help young children on Earth (e.g. those with immune or vascular dysfunction). Finally, if a multidisciplinary approach to provide pregnancy and postnatal care is developed for the isolated environment of spaceflight, this would have advantages for populations of women on Earth who live in remote areas. As these women may not be able to access antenatal and postnatal services regularly, this new telemedicine model of care could make a difference when applied on Earth.

## Summary and future outlook

The ESA SciSpacE white papers have been forward thinking by including human development and reproduction in its latest version. There are numerous benefits to conducting research in space, both for humans living and working in space, and for humans on Earth. Research in the fields of human development in space and reproductive medicine in space have many parallels, as progress in these fields ensures the ongoing and ever-developing needs of humans living and working in space are met. Whether humans chose to reproduce in space, knowledge gaps still exist that can impact how they live safely on Earth, after a spaceflight mission, and these need to be addressed. Highlighting sex-based differences related to all body systems is vital to ensure progressive and inclusive space research is conducted.
